# Supplementary material for: Identification of photoperiod-induced specific miRNAs in the adrenal glands of Sunite sheep (Ovis aries)
Source: Front Vet Sci. 2022 Jul 22;9:888207. doi: 10.3389/fvets.2022.888207 (PMC9354845; doi:10.3389/fvets.2022.888207)
Supplement: Supplementary file 1 [file Data_Sheet_1.ZIP › Supplementary file/miRDeep2 file.zip!/miRDeep2 file/Know_Structure/SPLP42A3/oar-mir-493.pdf]

miRBase precursor : oar-mir-493
Total read count : 11129
oar-miR-493-5p read count : 3153
oar-miR-493-3p read count : 7975
remaining reads : 1

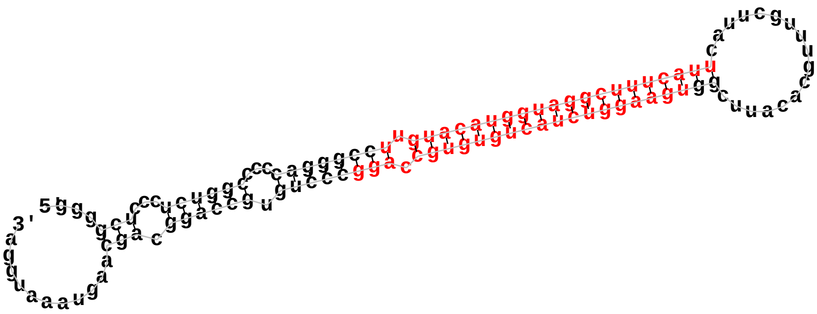

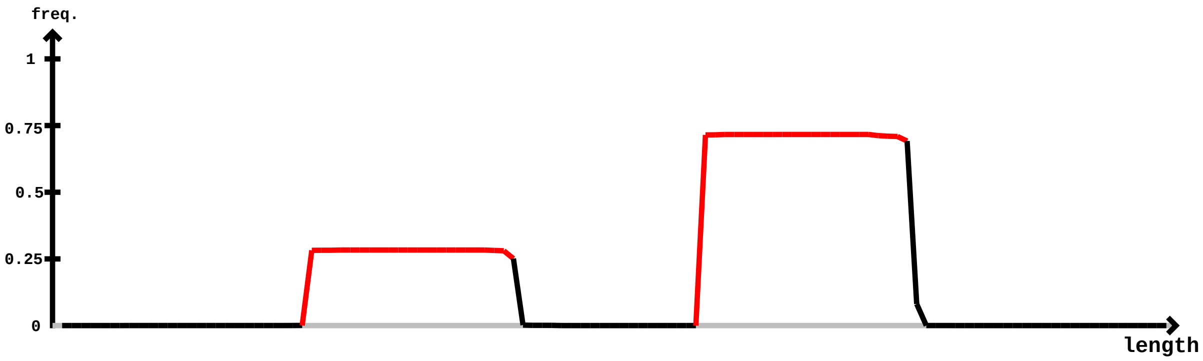

oar-miR-493-5p

oar-miR-493-3p

```
5'- ggggcucccucuggcccccagggccuuguacaugguaggcuuucauucauucguuugcacauucggugaaggucuacugugugccaggcccugugccaggcagcaaguaaaugga   -3'    exp
    ...(((...((((((...((((((((.((((((((((((((((((((.................)))))))))))))))))))).)))))))).)))))).)))...........   reads      mm         sample
    ......cccucuggcccccagggcc..........................................................................................   1          0          seq
    .......................ccuuguacaugguaggcuuuca......................................................................   1          0          seq
    ........................cuuguacaugguaggcuuuc.......................................................................   1          0          seq
    ........................cuuguacaugguaggcuuucauu....................................................................   3          0          seq
    .........................uuguacaugguaggcuuu........................................................................   1          0          seq
    .........................uuguacaugguaggcuuuc.......................................................................   16         0          seq
    .........................uuguacaugguaggcuuuca......................................................................   13         0          seq
    .........................uuguacaugguaggcuuucau.....................................................................   326        0          seq
    .........................uuguacaugguaggcuuucauu....................................................................   2770       0          seq
    .........................uuguacaugguaggcuuucauuc...................................................................   9          0          seq
    ..........................uguacaugguaggcuuucauu....................................................................   2          0          seq
    ..........................uguacaugguaggcuuucauuc...................................................................   1          0          seq
    ............................uacaugguaggcuuucauucau.................................................................   1          0          seq
    ............................uacaugguaggcuuucauucauu................................................................   9          0          seq
    ..................................................................ugaaggCcuacugugugc...............................   1          1          seq
    ..................................................................ugaaggucuacugugugc...............................   31         0          seq
    ..................................................................ugaaggucuacugugugU...............................   16         1          seq
    ..................................................................Agaaggucuacugugugc...............................   1          1          seq
    ..................................................................ugaaggCcuacugugugcc..............................   1          1          seq
    ..................................................................ugaaggucuacugugugcc..............................   22         0          seq
    ..................................................................ugaaggucuacugugugcca.............................   13         0          seq
    ..................................................................ugaaUgucuacugugugccag............................   2          1          seq
    ..................................................................ugaagAucuacugugugccag............................   1          1          seq
    ..................................................................ugaaggucuacugugugccag............................   185        0          seq
    ..................................................................ugaaggucuacugugugccagg...........................   6705       0          seq
    ..................................................................ugaagAucuacugugugccagg...........................   5          1          seq
    ..................................................................ugaaggCcuacugugugccagg...........................   23         1          seq
    ..................................................................ugaaggucuGcugugugccagg...........................   35         1          seq
    ..................................................................ugaaUgucuacugugugccagg...........................   4          1          seq
    ..................................................................ugaaggucuacuguguUccagg...........................   2          1          seq
    ..................................................................ugaaggucuacuAugugccagg...........................   9          1          seq
    ..................................................................ugaagguUuacugugugccagg...........................   3          1          seq
    ..................................................................ugaaggucuacugugugccaggc..........................   893        0          seq
    ..................................................................ugaaggCcuacugugugccaggc..........................   1          1          seq
```

```
                         oar-miR-493-5p
                                                                  oar-miR-493-3p
ggggcucccucuggcccccagggccuuguacaugguaggcuuucauucauucguuugcacauucggugaaggucuacugugugccaggcccugugccaggcagcaaguaaaugga

..................................................................ugaaggucuGcugugugccaggc..........................   3   1   seq
...................................................................gaaggucuacugugugccag............................   1   0   seq
...................................................................gaaggucuacugugugccagg...........................   2   0   seq
....................................................................aaggucuacugugugccagg...........................  11   0   seq
....................................................................aaggucuacugugugccaggc..........................   4   0   seq
..........................................................................uacugugugccaUgcccu.......................   1   1   seq
```
